# Supplementary material for: Preferential Detachment During Human Brain Development: Age- and Sex-Specific Structural Connectivity in Diffusion Tensor Imaging (DTI) Data
Source: Cereb Cortex. 2013 Dec 15;25(6):1477–89. doi: 10.1093/cercor/bht333 (PMC4428296; doi:10.1093/cercor/bht333)
Supplement: Supplementary Data [file supp_25_6_1477__index.html]

Preferential Detachment During Human Brain Development: Age- and Sex-Specific Structural Connectivity in Diffusion Tensor Imaging (DTI) Data — Preferential Detachment During Human Brain Development: Age- and Sex-Specific Structural Connectivity in Diffusion Tensor Imaging (DTI) Data — Supplementary Data 

# Preferential Detachment During Human Brain Development: Age- and Sex-Specific Structural Connectivity in Diffusion Tensor Imaging (DTI) Data

## Supplementary Data

Supplementary Data

**Files in this Data Supplement:**

- Supplementary Data - Doc file
- Supplementary Figure 1 - eps file
- Supplementary Figure 2 - eps file
- Supplementary Figure 3 - eps file
- Supplementary Figure 4 - eps file
- Supplementary Figure 5 - eps file
- Supplementary Figure 6 - eps file
