# Supplementary material for: Preferential Detachment During Human Brain Development: Age- and Sex-Specific Structural Connectivity in Diffusion Tensor Imaging (DTI) Data
Source: Cereb Cortex. 2013 Dec 15;25(6):1477–89. doi: 10.1093/cercor/bht333 (PMC4428296; doi:10.1093/cercor/bht333)
Supplement: Supplementary Data [file supp_bht333_bht333supp.doc]

**Supplementary Material**

**S1. Overall anatomical changes in brain volumes**

The overall anatomical changes in volume would inevitably affect topological and spatial features in the structural connectivity. Thus, before examining topological and spatial features of the structural connectivity in human developing brain, we explored volume changes. White matter volume (WMV), gray matter volume (GMV, cortical + subcortical) and intracranial volume (ICV) were tested with a general linear model (eq. 1-3, Table S1). All showed significant gender difference (*p* < 0.001). Males had larger volume than females did. WMV increased over age but the increasing rate slowed down after age 10 (*p* = 0.001), whereas GMV decreased significantly with increasing age (*β_1_* = -4171.981 mm^3^/year, *p* < 0.001), while ICV did not show any age effect (*p* = 0.069). In all cases, no significant gender and age interaction effect were observed so all models are fitted without the interaction term.

**Table S1.**  General Linear Model (GLM) analysis on white matter volume (WMV), gray matter volume (GMV) and intracranial volume (ICV):t values with degrees of freedom (*df*) and *p* values (two-sided, alpha 0.05) are provided in the table.

|  | Gender |  |  | Age |  |
| --- | --- | --- | --- | --- | --- |
|  | *t* (*df* =118) | *p* | | *t* (*df* =118) | *p* |
| WMV | -5.457 | < 0.001 | | 2.777 | 0.006 |
| GMV | -5.975 | < 0.001 | | -6.249 | < 0.001 |
| ICV | -6.776 | < 0.001 | | -0.418 | 0.677 |

**S2. Network Analysis**

**S2.1. Edge density**

The edge density represents the proportion of non-zero connections to the number of potential connections. Because our network is undirected, we used *d* = 2*E* / *N* (*N*-1), where *E* is the number of edges and *N* is the number of nodes. Note that the weights of individual edges might change but edge density will remain the same as long as the total number of edges is unchanged.

**S2.2. Global and local efficiency**

Global efficiency represents how well any two nodes of a network are connected, whereas local efficiency shows how well neighbours of a node are connected .


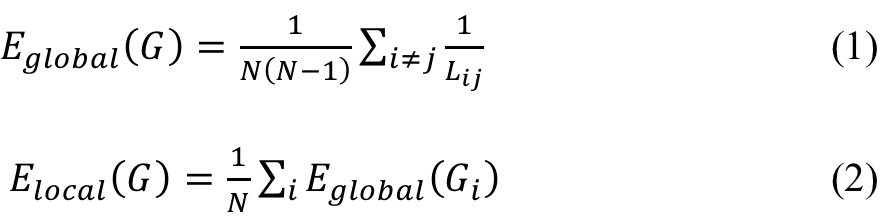


(S1)

(S2)

where *L*_i,j_: the length of the shortest path between nodes *i* and *j* (computed with Dijkstra algorithm), *N*: the number of nodes, *G*: a graph, *G_i_*: the subgraph that consists of neighbours of *i* without *i* itself.

Efficiency is greatly affected by the sparsity of the network ; when there are fewer edges and also even fewer streamlines, efficiency decreases. Thus we normalized efficiency with values obtained by 100 randomly rewired networks where randomly selected edges were swapped while preserving both degree and strength of each node . Whereas the number of connected edges of a node forms its degree, the strength of a node is the sum of weights of all its edges (here: total number of streamlines of that node) . Such a networks is called a small-world network when the local efficiency is much higher than in a comparable random network, but the global efficiency remains about the same.

**S2.3. Modularity**

We computed modularity *Q*, and estimated the modular membership, maximizing modularity , which may identify the functional blocks . Modularity measures the difference between the number of edges that lie within a community in the actual network (the first term) and a random network of the same degree sequence (the second term) for a certain membership assignment :


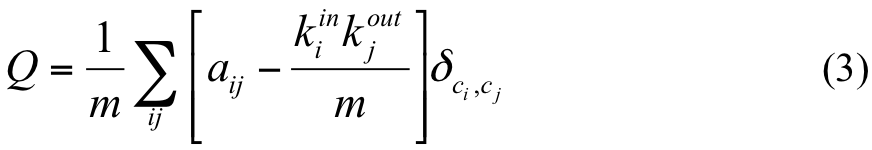


(S3)

where *m*: total number of streamlines in the network (note that bidirectional links are counted twice); *a*_ij_ : number of streamlines (weight) between node *i* and node *j*; *k*_i_^in^ : sum of streamlines in incoming edges of node *i*; *k*_j_^out^ : sum of streamlines in outgoing edges of node *j*; *δc*_i_*c*_j_ :Kronecker delta (only one if nodes *i* and *j* are in the same module and zero otherwise); *c*_n_ – label of module to which node *n* belongs to.

In addition to general linear model analysis across the entire age-range, we also grouped participants into five age-categories for modular membership analysis (Supp.Mat.S3 and Table S3). We tested the modular organization across age by matching module assignments of each participant to a representative participant whose average Normalized Mutual Information (NMI) is closest to that of all other participants in an age group . NMI quantifies how similar two modular structures are using information theory. We performed this procedure for each group and compared them by an omnibus test and also comparisons of consecutive groups.

We computed within-module strength and participation coefficient to examine nodal changes in modular structure. The within-module strength represents how well the node is connected to the others in the same module; high within-module strength implies that the node is more connected in the module where it belongs than the average connectivity of the other nodes in the module.

$$z_{i}=\frac{\kappa_{i}-\bar{\kappa}_{c_{i}}}{\sigma_{c_{i}}}$$

(S4)

where *κ_i_*: within-module strength of node i, *c_i_*: assigned modular membership, $\bar{k}_{c_{i}}$ and $\sigma_{c_{i}}$: average and standard deviation of within module strength of nodes in module *c_i_*.

Participation coefficient indicates how well the node is connected to all other modules. High participation coefficients index that the connections of a node are distributed to multiple modules. In extreme case, when the participation coefficient of a node is one, the node is connected to all the modules uniformly; and when it is zero, the node is solely connected to the nodes in its own module.

$$P_{i}=1-\sum_{s=1}^{N_{m}} \left(\frac{\kappa_{is}}{k_{i}}\right)^{2}$$

(S5)

where *k_i_*: degree (strength) of node i, *κ_is_*: number (sum of weights) of edges from node i to module s, *N_m_* is the number of modules.

**S3. Modular organization**

**Table S2. Participant statistics of five age groups.**

| Age range  [years] | Number of participants | Male/female | Age Mean (SD) |
| --- | --- | --- | --- |
| 4-11 | 13 | 6 / 7 | 8.31 (2.18) |
| 12-15 | 20 | 13 / 7 | 13.60 ( 0.99) |
| 16-19 | 14 | 7 / 7 | 17.43 (1.16) |
| 20-28 | 48 | 30 / 18 | 23.19 (2.46) |
| 29-40 | 26 | 13 / 13 | 34.19 (3.77) |

Note: For the membership assignment comparison, we grouped our participants into five age groups. The ratios between males and females were not significantly different between groups (χ^2^ test, χ^2^ (*df* = 4) = 2.539, *p* = 0.638).

**S3.1 Methods**

Following Alexander Bloch et al. , we estimated module assignment for each participant then matched to a representative participant whose average normalized Mutual Information (NMI) to the other participants were minimal in a group. The NMI quantifies how similar two modular structures are using information theory, given below

(S6)

where N is the number of nodes, C_A_ is the number of modules in structure A, C_B_ is the number of modules in structure B, N_i_ is the number of nodes in the i^th^ module of structure A, N_j_ is the number of nodes in the j^th^ modules of structure B, and Nij is the number of nodes which are intersection of the i^th^ module of structure A and the j^th^ module of structure B.

We performed this procedure for each group (Table 1). Matching algorithm maximized the overlap between modular structures. After matching the participants to the representative participants, we estimated most-frequently assignment for each node and compute the certainty as the ratio of the participants whose modular membership is the most-frequently-assignment to the number of total participants in the group.

In contrast to the previous methods : averaging of connectivity matrices of individuals before estimating modular structure; , averaging registered MRI scans before extracting networks), this method would 1) prevent the possibility that a spurious connectivity, which only exists in part of participants, may affect the modular structure and 2) provide how consistent modular membership were assigned to each node, showing inter-participant variability of modular membership assignment.

Alexander Bloch et al. also statistically tested regional differences in the modular membership between two groups, using the permutation test. For each node, they computed Pearson correlation of modular membership of the node between two participants, constructing similarity matrix of modular membership of the node. If there is a between-group difference, then the average within-group correlation would be higher than the between-group correlation. Using the permutation test (with 10,000 permutations), they sampled the distribution of average within-group correlations over permutations, and check where the true average within-group correlations was located to estimate p-value that the node’s membership assignment differs between groups.

In this study, with the Pearson correlation for modular membership of each node, we followed Kropf et al. to compare modular structure of multiple groups, because Alexander Bloch et al. ’s permutation test only works for two groups. Instead of estimating the distribution of average within-group correlation, Kropf et al. pursued the distribution of distances between average within-group correlation and average between-group correlation.

**S3.2 Uncorrected p-value results on nodal changes**

For five age groups, there were five regions whose uncorrected p-values are below the alpha level 0.05: left entorhinal (*p =* 0.008), left medial orbitofrontal (*p =* 0.023), left rostral anterior cingulate (*p =* 0.014), left postcentral (*p =* 0.041) and right postcentral ( *p =* -0.003). Interestingly they also showed significant age effect in the individual edge analysis (See table 1). The pair-wise comparison for each age group was also performed (Table S3).

Between males and females, there are eight regions whose uncorrected p-values are below the alpha level 0.05: left caudal middle frontal (*p =* 0.037), left medial orbitofrontal (*p =* 0.023), left pars triangularis (*p =* 0.023), left rostral anterior cingulate (*p =* 0.030), left rostral middle frontal (*p =* 0.045), left superior frontal (*p =* 0.024), left temporal pole (*p =* 0.003) and right insula (*p =* 0.047).

**S4. Possible scenarios to explain the reduction in the streamline count.**

We suggested our observation of a reduced number of streamlines might be explained by prolonged synaptic pruning concomitant with myelination increase (1). However, there are other possible explanations (2-5).

1) Decrease in the actual axonal projections from synaptic pruning or cell death

2) An edge (fibre tract) became more curved so could not be detected by our tractography algorithm (this is a well-known problem for DTI network reconstruction)

3) Less homogeneity of fibre directions🡪lower Fractional anisotropy🡪less likely detectable

4) Myelination reduce🡪 lower fractional anisotropy🡪less likely detectable

5) Axonal diameter enlarged 🡪lower fractional anisotropy🡪less likely detectable

Regarding (2), we cannot resolve the issue of limitation in finding highly curved fibres using DTI but we did test the role of the angular threshold for fibre tract reconstruction. The comparison between the original 35- degree and the more relaxed 45-degree criterion showed similar results confirming our earlier conclusions. While highly-curved fibers might still go undetected even with the relaxed criterion, we can test for the impact of curvature on our results by investigating whether edges characterised by a decreasing number of streamlines became more curved or not. If so, higher curvature streamlines that cannot be detected at old age might explain part of the loss of streamlines during development. On the other hand, if we do not find any changes in curvature of edges over age, we can conclude that the reduction in the streamline count cannot be explained by stronger curvature. Edges we tested did not show any systematic or consistent relationship between their curvatures and the age effects. Curvature of a fibre tract was estimated by dividing the trajectory length of fibre tract by the Euclidean distance between the centres of mass between a pair of nodes. Out of 128 edges, 21 edges showed significant curvature changes over age using GLM. Eleven edges were characterised by increased curvatures and 10 showed decreased curvatures. One edge with an increased curvature lost streamline count over age, with which we need to interpret the result with caution because it would be difficult to tell whether the decreased streamline count was from the higher curvature or from the loss of axonal projections. Interestingly, six fibre tracts with decreased curvature were also the edges that lost streamlines over age. These edges became straighter but lost streamline count over age, which can be stronger evidence showing the reduction in the number of streamlines was not from the changes in curvatures.

For (3): Less homogeneity of fibre directions, which means a smaller number (or a proportion) of streamlines with similar directions, or more crossing fibres. It is a possibility if we assume that axonal projections decrease over age because the reduction in the streamline count could change the proportions of fibre directions within voxels. However, the formation of maps between cortical areas, an on-going event during brain development, would increase the homogeneity of fibres . Homogeneity is also expected from anatomical findings of fibre lengths between two maps that are as short as possible

(4): Previous studies reported that myelination increases so we can exclude this possibility.

(5): Dilation of axon diameters is in principal possible. As the brain size enlarges, axonal fibres might lengthen and to obtain the same conduction velocity axons may need to widen their diameters or increase myelination or both. In our study, we did not observe any significant lengthening of fibre tracts even more stabilised after 10 year old (Spearman's rho: 0.1704, *p* = 0.0617). Therefore, this scenario is less likely to explain the overall reduction in the streamline count. “

**S5. The relationship between the length of a fibre tract and its weight (streamline count)**

Brain is connected in a way that the total wiring length is optimally short with a few long-distance connections, which benefits efficient communications between brain regions while spends less amount of resourses . Likewise, to shorten to total connection length, long fibre tracts may consist of fewer number of streamlines than short-range ones. However, the tendency was not a simple negative correlation; short edges showed a wide spectrum of different weights and ‘thick’ fibre tracts with many streamlines could be long too (Figure S4A). Thus long fibre tracts are not always ‘thinner’ edges. In addition, there were more thicker and shorter edges within modules than between modules but they showed much overlapping areas in the scatterplot (Figure S4B) indicating within module edges are not always short and between module fibre tracts can be also short. Within-module fibre tracts were comprised of more short edges than between-module edges in general (Figure S4B inset) but not necessarily short. We found that only 47% of inter-modular fibre tracts also belongs to the class of long-distance connections and 43% of thin fibre tracts were also long distance-connections.Therefore, we analysed all three types of fibre tracts independently and connected with small-world topology, modular organization and edge density, respectively in the main text.

**S6. Individual edge analysis with different parameters for tractography.**

We investigated whether various parameters for tractography would also bring consistent results using a wider angular threshold of 45 degrees and 10 random seeds tracking per voxel instead of a single seed point at the centre. Figure S5 displays the results of individual edge analysis using 4 different settings. Visual inspection shows the results were consistent across conditions with few differences. Fibre tracts with a decreased number of streamlines showed 84 to 90 % overlap and fibre tracts with an increased number of streamlines demonstrated 0 to 67% consistency and edges with sex-specific effects were also consistently marked as significant from 50% to 100 %. Note that we only compared our results and three different conditions so 6 comparisons in total. It appeared that a wider angular threshold brought the most difference but still 35 degrees and 45 degrees with a single seed displayed quite similar results (compare Figure S5A and S5C). Ten random seeds per voxel contributed more consistent results between sexes, thus less number of edges with interaction effects between age and sex, which supports our hypothesis of a delayed but similar development between genders (refer to Figure 6).

**Table S3. Pair-wise comparison of modular organization (uncorrected p-values)**

| Age groups | 4-11 vs. 12-15 | 14-15 vs. 16-19 | 16-19 vs. 20-28 | 20-28 vs. 29-40 |
| --- | --- | --- | --- | --- |
|  | *P =* 0.1561  lh.medialorbitofrontal: *p =* 0.035  lh.inferiorparietal: *p =* 0.043  lh.superiorparietal: *p =* 0.043  rh.parahippocampal: *p =* 0.044  rh.paracentral: *p =* 0.048  rh.postcentral: *p =* 0.005  rh.posteriorcingulate: *p =* 0.025 | *P =* 0.2140  lh.medialorbitofrontal: *p =* 0.031  lh.superiorfrontal: *p =* 0.049  rh.inferiorparietal: *p =* 0.025  rh.precentral: *p =* 0.049 | *P =* 0.3414  lh.medialorbitofrontal: *p =* 0.046  lh.postcentral: *p =* 0.022  rh.bankssts: *p =* 0.046  rh.caudalmiddlefrontal: *p =* 0.023  rh.postcentral: *p =* 0.024  rh.transversetemporal: *p =* 0.019  rh.amygdala: *p =* 0.017  rh.hippocampus: *p =* 0.048 | *P =* 0.1119  lh.medialorbitofrontal: *p =* 0.008  lh.bankssts: *p =* 0.045  lh.caudalanteriorcingulate: *p =* 0.018  lh.entorhinal: *p =* 0.039  lh.lingual: *p =* 0.043  lh.parahippocampal: *p =* 0.013  lh.parsopercularis: *p =* 0.028  lh.precentral: *p =* 0.039  lh.rostralanteriorcingulate: *p =* 0.002  lh.superiorfrontal: *p =* 0.009  rh.caudalmiddlefrontal: *p =* 0.006  rh.parsopercularis: *p =* 0.020  rh.rostralanteriorcingulate: *p =* 0.035  rh.rostralmiddlefrontal: *p =* 0.026  rh.superiorfrontal: *p =* 0.032 |

**Table S4. Fibre tracts with age-related changes for both genders.**

| **ROI (node)** | **Lobe** | **ROI (node)** | | **Lobe** | **Slope** | **FDR adjusted P** |  |
| --- | --- | --- | --- | --- | --- | --- | --- |
| lh.bankssts | T | lh.middletemporal | | T | -0.941 | 0.001 |  |
| lh.caudalanteriorcingulate | F | rh.caudalanteriorcingulate | | F | -0.487 | 0.017 | Inter-hemispheric |
| lh.caudalanteriorcingulate | F | lh.superiorfrontal | | F | -1.542 | 0.024 | Symmetric |
| lh.caudalmiddlefrontal | F | lh.precentral | | F | -1.458 | 0.016 |  |
| lh.caudalmiddlefrontal | F | lh.superiorfrontal | | F | -1.26 | <10 ^-4^ | Symmetric |
| lh.cuneus | O | lh.precuneus | | P | -1.488 | 0.002 | Symmetric |
| lh.fusiform | T | lh.inferiortemporal | | T | -0.356 | 0.043 | Symmetric |
| lh.isthmuscingulate | P | lh.precuneus | | P | -0.726 | 0.016 |  |
| lh.lateralorbitofrontal | F | lh.insula | |  | -2.008 | 0.001 | Symmetric |
| lh.medialorbitofrontal | F | rh.medialorbitofrontal | | F | -1.03 | 0.0003 | Inter-hemispheric |
| lh.middletemporal | T | lh.superiortemporal | | T | -0.513 | 0.016 | Symmetric |
| lh.paracentral | F | lh.precuneus | | P | -2.213 | <10 ^-5^ | Symmetric |
| lh.paracentral | F | lh.posteriorcingulate | | P | -0.671 | 0.016 | Symmetric |
| lh.parsopercularis | F | lh.parstriangularis | | F | -0.343 | 0.014 | Symmetric |
| lh.parsopercularis | F | lh.precentral | | F | -2.269 | <10 ^-5^ | Symmetric |
| lh.parsopercularis | F | lh.rostralmiddlefrontal | | F | -0.455 | 0.004 | Symmetric |
| lh.parsorbitalis | F | lh.parstriangularis | | F | -1.077 | 0.016 |  |
| lh.postcentral | P | lh.supramarginal | | P | -0.381 | 0.041 |  |
| lh.posteriorcingulate | P | lh.superiorfrontal | | F | -1.285 | 0.01 |  |
| lh.posteriorcingulate | P | rh.posteriorcingulate | | P | -1.303 | 0.003 | Inter-hemispheric |
| lh.precuneus | P | rh.precuneus | | P | -0.599 | 0.002 | Inter-hemispheric |
| lh.precuneus | P | lh.superiorparietal | | P | -0.504 | 0.012 |  |
| lh.putamen |  | lh.pallidum | |  | -1.02 | 0.009 | Subcortical |
| lh.rostralanteriorcingulate | F | lh.superiorfrontal | | F | -1.63 | 0.012 | Symmetric |
| lh.rostralmiddlefrontal | F | lh.superiorfrontal | | F | -1.275 | 0.0003 | Symmetric |
| lh.superiortemporal | T | lh.supramarginal | | P | -1.725 | 0.004 | Symmetric |
| rh.bankssts | T | rh.superiortemporal | | T | -0.688 | 0.001 |  |
| rh.caudalanteriorcingulate | F | rh.superiorfrontal | | F | -1.081 | 0.0004 | Symmetric |
| rh.caudalmiddlefrontal | F | rh.superiorfrontal | | F | -0.422 | 0.024 | Symmetric |
| rh.cuneus | O | rh.precuneus | | P | -0.426 | 0.021 | Symmetric |
| rh.fusiform | T | rh.inferiortemporal | | T | -0.725 | 0.004 | Symmetric |
| rh.fusiform | T | rh.lateraloccipital | | O | -0.161 | 0.045 |  |
| rh.fusiform | T | rh.parahippocampal | | T | -2.39 | 0.002 |  |
| rh.inferiorparietal | P | rh.lateraloccipital | | O | -1.131 | 0.001 |  |
| rh.lateraloccipital | O | rh.superiorparietal | | P | -0.657 | 0.007 |  |
| rh.lateralorbitofrontal | F | rh.parsorbitalis | | F | -0.794 | 0.001 |  |
| rh.lateralorbitofrontal | F | rh.insula | |  | -0.616 | 0.016 | Symmetric |
| rh.medialorbitofrontal | F | rh.superiorfrontal | | F | -0.879 | 0.01 |  |
| rh.middletemporal | T | rh.superiortemporal | | T | -0.54 | 0.001 | Symmetric |
| rh.paracentral | F | rh.posteriorcingulate | | P | -0.481 | 0.017 | Symmetric |
| rh.paracentral | F | rh.precuneus | | P | -0.873 | 0.001 | Symmetric |
| rh.parsopercularis | F | rh.parstriangularis | | F | -1.05 | 0.0003 | Symmetric |
| rh.parsopercularis | F | rh.precentral | | F | -0.313 | 0.04 | Symmetric |
| rh.parsopercularis | F | rh.insula | |  | -0.321 | 0.016 |  |
| rh.parstriangularis | F | rh.rostralmiddlefrontal | | F | -2.066 | <10 ^-5^ | Symmetric |
| rh.postcentral | P | rh.superiorparietal | | P | -0.613 | 0.0002 |  |
| rh.postcentral | P | rh.supramarginal | | P | -0.88 | 0.002 | Symmetric |
| rh.posteriorcingulate | P | rh.superiorfrontal | | F | -0.497 | 0.017 |  |
| rh.precentral | F | rh.insula | |  | -2.379 | <10 ^-5^ |  |
| rh.rostralanteriorcingulate | F | rh.superiorfrontal | | F | -0.232 | 0.016 | Symmetric |
| rh.rostralmiddlefrontal | F | rh.superiorfrontal | | F | -0.793 | 0.016 | Symmetric |
| rh.superiortemporal | T | rh.supramarginal | | P | -0.56 | 0.046 | Symmetric |
| rh.supramarginal | P | rh.transversetemporal | | T | -1.279 | 0.0004 |  |
| rh.temporalpole | T | rh.insula | |  | -0.254 | 0.007 |  |
| rh.transversetemporal | T | rh.insula | |  | -2.329 | <10 ^-5^ |  |
| F:50 | P:27 |  | | T:20 | O:5 |  |  |
| lh.caudalanteriorcingulate | F | lh.posteriorcingulate | | P | 0.377 | 0.012 |  |
| rh.isthmuscingulate | P | rh.posteriorcingulate | | P | 0.456 | 0.034 |  |
| F:1 | P:3 | |  | T:0 | O:0 |  |  |

Note: **Decreased connections:** Interhemispheric connections and a subcortical fiber tract showing a decreased number of streamlines are bolded. **Increased connections (the penultimate two rows):** The last row of each section gives an overview of how often different lobes participate in these changes. (F: Frontal lobe, P: Parietal lobe, T: Temporal lobe, O: Occipital lobe). P values were adjusted by FDR with a q level 0.05 .

**Table S5. Abbreviation of ROI names**

| **Abbreviated name** | **Full name** |
| --- | --- |
| BSTS.  CAC  CMF.  CUN  ENT  FUS  IP.  IT.  ISTC  LOCC  LOF.  LING.  MOF  MT.  PARH  PARC  POPE  PORB  PTRI  PCAL  PSTC  PC  PREC  PCUN -  RAC  RMF  SF  SP  ST  SMAR  FP  TP  TT  INS  THAL  CAUD  PUTA  PAL  AMYG  HIPP  ACC | Banks of the superior temporal sulcus  caudal anterior cingulate  caudal middle frontal  cuneus  entorhinal  fusiform  inferior parietal  inferior temporal  isthmus of the cingulate  lateral occipital  lateral orbitofrontal  lingual  medial orbitofrontal  middle temporal  parahippocampal  paracentral  pars opercularis  pars orbitalis  pars triangularis  peri-calcarine  postcentral  posterior cingulate  precentral  precuneus  rostral anterior cingulate  rostral middle frontal  superior frontal  superior parietal  superior temporal  supra-marginal  frontal pole  temporal pole  transverse temporal  insula  thalamus  caudate  putamen  pallidum  amygdala  hippocampus  accumbens |

---------------------------------------Insert Figure S1 here--------------------------------------

**Figure S1. Modular structure over four age groups** (shown table S2)**:** 4-11 y/o (A & E), 12-15 y/o (B & F), 16-19 y/o (C & G), and 20-28 y/o (D & H). For each group, we selected the representative participant which showed the smallest average normalized mutual information (NMI), and matched all the other participants to the representative participant, maximizing overlaps between modular memberships. Then we counted the most frequently occurred community membership over each group (upper row, A, B, C, and D) and showed the ratio of the most-frequently occurred community membership to the number of participants in each group as a certainty of averaging (within-group certainty, bottom row, E, F, G, and H). In the upper row, the community structure is largely unchanged with left anterior (blue), left posterior (white), right anterior (red), and right posterior (yellow) modules. . The left anterior module (red) was located on the frontal lobe and extended to the parietal lobe and temporal lobe, while left posterior module (yellow) resided in the left occipital and parietal lobes. Right anterior module (blue) in the frontal lobe and extended to the right temporal lobe occasionally, while right posterior (white) resides mostly in the occipital lobe and extended to the parietal and temporal lobes. The left central module (green) was very small in the youngest age group (two nodes). In the bottom row, the whiter circles represent more certain modular membership assignment, where red circle showed the nodes whose average certainty is below 50%. In the modular organization of the youngest group A, there were only two nodes in the green module but it does not mean that there were only two nodes in the particular module. The number of nodes in the green module varied from 0 to 13; there were very high variability thus having very low certainty (E); consistent results were only for two nodes but the module is much larger for each individual member of that age group.

---------------------------------------Insert Figure S2 here--------------------------------------

**Figure S2. Streamline count range. A.** Streamline count ranges for individual edges (Male) **B.** Streamline count ranges for individual edges (Female), 128 tested edges shared by all participants with box plots. Red cross: outliers.

---------------------------------------Insert Figure S3 here--------------------------------------

**Figure S3. Sex difference in the individual edges.** Gender differences were observed mostly within modules and only within hemispheres. (Gray: edges connecting ROIs within modules and Light gray: edges between modules, both with no significant change over age; Red: edges with a larger number of streamlines for males than females; Blue: edges with a larger streamline count for females than males).

---------------------------------------Insert Figure S4 here--------------------------------------

**Figure S4. The relationship among between the average length of an edge and its weight (streamline count)** A. Scatterplot. Darker areas have more data points. Short fibre tracts tend to have larger number of streamlines and the fibre tract with many streamlines (‘thick’) is more likely to be short-range. However, the tendency was not a simple negative correlation (Supp.Mat S5). B. Scatterplot differentiating intra- and inter-module fibre tracts. Inset: the probability density estimate for fibre tracts according to lengths using kernel smoothing density. Within module fibre tracts were comprised of more short edges than between module edges. Each line represents a participant. Red: edges within modules, Blue: edges between modules.

---------------------------------------Insert Figure S5 here--------------------------------------

**Figure S5. Individual edge analysis with 35 and 45 degrees of angular threshold and single and ten random seeds tracking.** First row: 35 degrees of angular threshold, Second row: 45 degrees of angular threshold. Left column: a single seed at the centre per voxel, Right column: ten random seeds tracking per voxel.

---------------------------------------Insert Figure S6 here--------------------------------------

**Figure S6. Slopes with FDR adjusted confidence intervals for the edges with age effects for both genders with four different tracking parameters. .** First row: 35 degrees of angular threshold, Second row: 45 degrees of angular threshold. Left column: a single seed at the centre per voxel, Right column: ten random seeds tracking per voxel. Slopes are represented as sorted and different tracking parameters resulted in different number of testable edges that all participants shared, leading to slightly different results. The result with 10 random seeds tracking demonstrated approximately 10-fold slopes than the resulting slopes with the original parameters (35 degrees with a single tracking per voxel).

**References**

Achard S, Bullmore E (2007) Efficiency and cost of economical brain functional networks. PLoS Comput Biol 3: e17.

Alexander-Bloch A, Lambiotte R, Roberts B, Giedd J, Gogtay N, Bullmore E (2012) The discovery of population differences in network community structure: New methods and applications to brain functional networks in schizophrenia. NeuroImage 59: 3889-3900.

Alexander-Bloch A, Lambiotte R, Roberts B, Giedd J, Gogtay N, Bullmore E (2012) The discovery of population differences in network community structure: new methods and applications to brain functional networks in schizophrenia. Neuroimage 59: 3889-3900.

Barrat A, Barthelemy M, Pastor-Satorras R, Vespignani A (2004) The architecture of complex weighted networks. Proc Natl Acad Sci U S A 101: 3747.

Benjamini Y, Hochberg Y (1995) Controlling the false discovery rate: a practical and powerful approach to multiple testing. Journal of the Royal Statistical Society Series B (Methodological): 289-300.

Benjamini Y, Yekutieli D, Don E, Shaffer JP, Tamhane AC, Westfall PH, Holland B (2005) False Discovery Rate: Adjusted Multiple Confidence Intervals for Selected Parameters [with Comments, Rejoinder]. Journal of the American Statistical Association 100: 71-93.

Bullmore E, Sporns O (2012) The economy of brain network organization. Nature Reviews Neuroscience 13: 336-349.

Chklovskii DB, Koulakov AA (2004) Maps in the brain: What can we learn from them? Annu Rev Neurosci 27: 369-392.

Duarte-Carvajalino JM, Jahanshad N, Lenglet C, McMahon KL, de Zubicaray GI, Martin NG, Wright MJ, Thompson PM, Sapiro G (2011) Hierarchical topological network analysis of anatomical human brain connectivity and differences related to sex and kinship. Neuroimage.

Fair DA, Cohen AL, Power JD, Dosenbach NU, Church JA, Miezin FM, Schlaggar BL, Petersen SE (2009) Functional brain networks develop from a "local to distributed" organization. PLoS computational biology 5: e1000381.

Guimera R, Amaral LA (2005) Cartography of complex networks: modules and universal roles. Journal of statistical mechanics (Online) 2005: nihpa35573.

Jung K, Friede T, Beißbarth T (2011) Reporting FDR analogous confidence intervals for the log fold change of differentially expressed genes. BMC bioinformatics 12: 288.

Kaiser M (2011) A Tutorial in Connectome Analysis: Topological and Spatial Features of Brain Networks. Neuroimage 57: 892-907.

Kaiser M, Hilgetag CC (2006) Nonoptimal Component Placement, but Short Processing Paths, due to Long-Distance Projections in Neural Systems. PLoS Computational Biology 2: e95.

Kropf S, Heuer H, Gruning M, Smalla K (2004) Significance test for comparing complex microbial community fingerprints using pairwise similarity measures. Journal of microbiological methods 57: 187-195.

Latora V, Marchiori M (2001) Efficient behavior of small-world networks. Physical Review Letters 87: 198701.

Leicht EA, Newman ME (2008) Community structure in directed networks. Phys Rev Lett 100: 118703.

Newman ME (2006) Modularity and community structure in networks. Proc Natl Acad Sci U S A 103: 8577-8582.

Rubinov M, Sporns O (2011) Weight-conserving characterization of complex functional brain networks. NeuroImage.

von der Malsburg C (1973) Self-Organization of Orientation Sensitive Cells in the Striate Cortex. Kybernetik 14: 85-100.

Watts DJ, Strogatz SH (1998) Collective Dynamics of 'small-World' Networks. Nature 393: 440-442.
